# Academic education of midwives in Germany (part 1): Requirements for bachelor of science programmes in midwifery education. Position paper of the Midwifery Science Committee (AHW) in the DACH Association for Medical Education (GMA)

**DOI:** 10.3205/zma001688

**Published:** 2024-06-17

**Authors:** Claudia F. Plappert, Nicola H. Bauer, Kirsten Dietze-Schwonberg, Melita Grieshop, Annette Kluge-Bischoff, Birgit-Christiane Zyriax, Sabine Striebich

**Affiliations:** 1 University of Tübingen, Medical Faculty, Institute of Health Sciences, Tübingen, Germany; 2 University of Cologne and University Hospital Cologne, Medical Faculty, Institute for Midwifery Science, Cologne, Germany; 3 University Medical Center of Johannes Gutenberg University Mainz, Mainz, Germany; 4 Protestant University of Applied Sciences Berlin, Berlin, Germany; 5 University of Augsburg, Medical Faculty, Augsburg, Germany; 6 University Medical Center Hamburg-Eppendorf, Midwifery Science – Health Services Research and Prevention, Institute for Health Services Research in Dermatology and the Nursing Professions (IVDP), Hamburg, Germany; 7 Martin-Luther-University Halle-Wittenberg, University Medicine Halle, Medical Faculty, Institute for Health and Nursing Science, Halle (Saale), Germany

**Keywords:** academisation, healthcare professions, midwives, curricula

## Abstract

The current situation in Germany is characterised by significant differences between the two types of higher education institutions offering bachelor's degree programmes in midwifery at both universities of applied sciences and universities. These differences are noticeable in admission procedures, resource allocation, content focus and competence assessment at the respective institutions, which in turn result in heterogeneous study experiences. This article highlights the challenges currently facing bachelor degree programmes and the academic qualification of midwives, and identifies future requirements for the development of degree programmes in theory and practice as well as theory-practice transfer, and assessment formats.

Furthermore, this article covers the content-related and structural-organisational requirements to develop in-depth academic skills grounded in theory teaching, the facilitation of clinical placements at an academic level, the training of qualified practical instructors and the development of applicable competence-based assessment formats, especially for the state exam.

The development of a standardised, high-quality academic education for midwives in Germany requires networking of the different academic sites/locations to exchange experiences in teaching/learning and assessment formats. Furthermore, it can facilitate the development of a standardised competence-oriented model and core curriculum as well as the definition of quality criteria and standards for study programmes of midwifery science. The Midwifery Science Committee (AHW) in the DACH Assoviation for Medical Education (GMA) offers an optimal platform for cooperation between the different universities. The existing challenges for the further professional development of midwives can only be overcome by collaboration and pooled expertise.

## Abstract (de)

An den HAWs und universitären Standorten für hebammenwissenschaftliche Bachelor-Studiengänge in Deutschland bestehen derzeit große Unterschiede hinsichtlich der Zulassungsverfahren, Ressourcenausstattung und inhaltlichen Schwerpunktsetzungen, was zu heterogenen Curricula und Lehrangeboten führt. Der vorliegende Artikel zeigt auf, welche Herausforderungen für die Hochschulen und für die akademische Qualifizierung von Hebammen bestehen und benennt zukünftige Erfordernisse an die Studiengangsentwicklung in Theorie und Praxis, im Theorie-Praxis Transfer und an Prüfungsformate.

Aufgezeigt werden sowohl inhaltlich-konzeptionelle als auch strukturell-organisatorische Anforderungen, wie z.B. benötigte Lehrformate zur Entwicklung tiefgreifender wissenschaftlicher Kompetenzen in der theoretischen Lehre, die Realisierung der praktischen Studienphasen auf akademischem Niveau, die Ausbildung qualifizierter Praxisanleitenden sowie die Entwicklung geeigneter kompetenzorientierter Prüfungsformate insbesondere für die staatliche Prüfung.

Für die Entwicklung einer einheitlichen hochwertigen akademischen Bildung von Hebammen in Deutschland bedarf es der Vernetzung der Studienstandorte zum Austausch von Erfahrungen zu Lehr-/Lern- und Prüfungsformaten, der Entwicklung eines einheitlichen Kompetenzstrukturmodells und Kerncurriculums sowie der Definition von Qualitätskriterien und Standards für das Studium der Hebammenwissenschaft. Der Ausschuss Hebammenwissenschaft in der Gesellschaft für Medizinische Ausbildung bietet eine ideale Plattform für die Zusammenarbeit der Studienstandorte. Nur in gemeinschaftlicher Arbeit und mithilfe gebündelter Expertisen können die bestehenden Herausforderungen für die weitere Professionsentwicklung von Hebammen bestmöglich bewältigt werden.

## 1. Introduction

This article written by the Midwifery Science Committee (AHW) in the DACH Association for Medical Education (GMA) outlines challenges universities in Germany are facing as part of the transition to the academic training of midwives. Part two highlights tasks for development for the midwifery profession in Germany as a result from both the new law for the profession and the health policy framework.

## 2. Background

Until 2020, midwives went through a vocational training to obtain a midwifery diploma. However, the increasingly complex demands of the midwifery profession require extended professional skills that were not sufficiently reflected in the subject-based training under the Midwifery Act of 1985 [1] and the Midwifery Training and Examination Ordinance of 1987 [2]. In November 2019, the amendment to the Midwifery Act paved the path for the full academisation of the profession [3]. The transition to academic training implemented followed both the recommendation made by the Scientific Commission of the German Science and Humanities Council (WR) in 2012 [4], [5] and the EU Directives 2005/36/EC as well as the directive 2013/55/EU on the automatic recognition of professional qualifications in Germany. In doing so, Germany follows developments in Austria and Switzerland, where the academisation of midwifery training was implemented in 2006 and 2008 respectively.

1. **The situation and challenges for midwifery study sites in Austria: A brief overview**
  In 2006, Austria introduced Bachelor's degree proprammes, which, as usual at Austrian universities of applied sciences, consist of six semesters and award 180 ECTS credits. Today, there are study locations in eight of the nine federal states. These programmes are planned based on the Midwifery Act (HebG) and the University of Applied Sciences Studies Act (FHStG) in collaboration with the corresponding implementation ordinance (FH-Heb-AV). Eventually, they are accredited by the Agency for Quality Assurance and Accreditation. In defining what are known as minimum standards, the FH Heb-AV is less differentiating than its German equivalent, the HebStPrV. This is reflected, for instance, in the competence orientation, evidence-based approach as well as the qualification, and the level of practical guidance. This gives universities of applied sciences in Austria more authority with regard to the specific design and focus of the degree programmes. The midwifery degree programme in Austria is not planned as a dual course. Therefore, it does not require students to have one identified cooperating practice partner (usually a hospital). This again makes it easier to gain a wide range of practical experience in different settings, as well as placements abroad. Adjustments to publicly funded study places are calculated on the grounds of corresponding requirement analysis and funding from the federal states. The creation of ongoing postgraduate academic career pathways for midwives in line with the Bologna Process (Master’s, doctorate, habilitation) is not yet implemented in Austria. An exception to this are a few continuing education Master's degree programmes in Midwifery Science (in agreement with the Austrian Higher Education Legislation Package 2021). Midwifery science Master’s degrees are not publicly funded. Those who are interested must finance the fees of approx. 11,000 EUR themselves. There are currently no institutionalized doctoral programmes in Austria. Therefore, if midwives are admitted, doctoral studies are often associated with high tuition fees.
2. **The situation and challenges for midwifery study sites in Switzerland: A brief overview**
  The midwifery profession in Switzerland is a full academic training since 2008. Western Switzerland (Haute école de santé, Genève) led the way in 2002 by introducing a Bachelor's degree course for midwives. In 2008, German-speaking Switzerland followed with two study locations in Bern and Zurich (BFH, ZHAW). The BSc in Midwifery degree programmes are structured over six semesters and are accredited with 180 ECTS, as is standard at universities of applied sciences in Switzerland. To date, there is no undergraduate midwifery degree programme at a university. Planning is based on the Health Professions Act (GesBG, 2020), and the associated ordinances (Health Professions Competence Ordinance, Health Professions Recognition Ordinance, Ordinance on the Accreditation of Study Programmes, Register Ordinance). In similarity to Austria, the requirements in Switzerland are less detailed than the legal foundations HebG and HebStPrV in Germany. For the standardized and obligatory final competencies as defined by the federal government for the health professions, professionspecific models were derived following the so-called “CanMeds”-framework for the university of applied sciences professions. Future midwives have to complete a 10-months long practice placement before they start studying or after completing the sixth semester to achieve qualification for the profession. This provides the needed practice hours, which cannot be completed within a 6-semester course. During these placements, students are accompanied by practice trainers/mentors in hospitals. The first revision of the curriculum took place in 2018-20 and the competencies for the CanMeds framework were revised. In order to ensure that a sufficiently large number of midwives are trained for Switzerland, the number of study locations was increased. Since then, the most significant challenge in this connetx, was to provide an adequate number of locations for practice placements for students.

The recently enacted Midwifery Act aims to empower midwives to work in line with evidence-based and autonomous principles [6]. The legal reform has fundamentally changed the training of midwives in Germany. The theoretical framework significantly expanded to include principles of scientific research, medical ethics as well as the development of both complex care plans and personnel skills. In addition, clinical placements were shortened and interlinked with theoretical content. Moreover, clinical experience in the community was extended and a proportion of 25% practice skills training by named mentorship midwives was stipulated mandatory.

The main features and requirements of degree programmes are defined in the Midwifery Act (HebG) [https://www.gesetze-im-internet.de/hebg_2020/BJNR175910019.html] and in the Midwifery Study and Examination Ordinance (HebStPrV) [https://www.gesetze-im-internet.de/hebstprv/BJNR003900020.html]. The aim of the study programme is to facilitate essential professional and personal skills (§ 9 HebG). The modified competence profile of the International Confederation of Midwives (ICM) forms the foundation of the HebStPrV competence profile listed in attachment 1 [https://www.gesetze-im-internet.de/hebstprv/BJNR003900020.html]. These competencies must be assessed as part of the three-part state exam (written, oral, clinical practice).

The dual midwifery degree programme raises the level of the previous vocational training in the German Qualifications Framework (Deutschen Qualifikationsrahmens – DQR) [7] from level four to level six. The DQR is an assessment tool that describes competence levels, which are necessary to qualify in a particular field and translates the European Qualifications Framework (EQF) into national legislation. This again ensures the implementation of the Bologna reform [6]. 

The competence description at level six fulfils the current complex requirements of the midwifery profession. The scope of midwifery practice encompasses the entirety of the maternal and parental life cycle, from family planning through pregnancy, birth, the postnatal period, and breastfeeding until the end of the child's first year or until the end of the breastfeeding period (referred to as the “midwifery care continuum”) [8]. Therefore, the graduate midwife is the healthcare professional who is qualified to provide all healthcare for women (The term “woman” refers to the biological female sex and is independent of a person’s gender identity) throughout a large part of the reproductive phase [9]. The target group also includes individuals who identify as trans*, inter, or non-binary and do not self-identify as women. In the following section, the gender identity of individuals who utilise midwifery services is not explicitly identified through the use of linguistic markers, but is instead referred to as women. 

In Germany, midwifery education can take place in different academic settings, including universities of applied sciences (HAW), traditional universities and in joint initiatives between these two institutions. Dual study programmes are distinguished by two key characteristics: Firstly, the teaching content is realised at two coordinated learning locations, a university and a company. Secondly, the orientation is towards the primacy of academia [10]. Currently (as of April 2024), 48 university of applied sciences and universities across Germany offer a dual study programme for student midwives [11]. They combine a professional certificate as a midwife and a bachelor of science degree in midwifery science. All institutions of higher education offering degrees in midwifery science are characterised by a commitment to apply the rigours of scientific enquiry to clinical practice. The curriculum content is not only presented in lectures but also at cooperating healthcare institutions. This in turn ensures that skills acquisition in all clinical settings is both evidence- and best practice-based. Midwifery science degree programmes are categorised as dual practice-integrated, incorporating clinical phases within the curriculum [12]. 

The disparities in resources and content emphasis between HAWs and universities result in a heterogeneity of curricula, teaching content as well as admission procedures [13], [14]. The process of selecting students for placement varies depending on the institution. At some institutions, students are selected by the university and then placed with one of the practice partners. At others, students apply for a practical placement and only apply to the university once they have been accepted by practice partners. In order to promote long-term development of the midwifery profession, it is essential that the different study locations establish common goals, such as the inauguration of core teaching content in the curricula. An alignment between the institutions of higher education will facilitate the implementation of the midwifery profession in Germany and promote its successful development.

## 3. Requirements for practice-integrated study programmes

The duration of the full-time degree programme takes a minimum of six and a maximum of eight semesters, as stipulated in § 11 HebG. The dual study programme comprises a university-based theoretical and a clinical component, both of which must have a minimum scope of 2,200 hours. The total study time must comprise at least 4,600 hours. All clinical placements must include 25 % clinical practice under the supervision of a trained mentor midwife (§ 13 (2)). 

### 3.1. Theoretical training

In line with the Bologna reform to introduce Bachelor’s and Master’s degree programmes across Europe and accreditation requirements in Germany, midwifery science degree programmes focus on competency-based education. There are six clearly defined competence areas in which students must demonstrate their proficiency in order to successfully complete the programme. They include:

1. independent and evidence-based support and management of physiological processes during pregnancy, birth, postpartum and breastfeeding;
2. planning, organising, implementing, managing and evaluating of complex care plans;
3. promoting of women's autonomy and empowerment of self-determination during pregnancy, birth, postpartum and breastfeeding;
4. communication and
5. intra and interprofessional collaboration as well as
6. reflection and justification of own actions (see attachment 1 , table S1).

In order to fulfil these competencies, it is important for curricula to be grounded in scientific principles and evidence based medicine [15]. As part of the academic training, the ratio of theory to practice was increased. Furthermore, lectures are held by an interprofessional team to give students the specific skills they need to meet the demanding realities of maternity care [https://www.gesetze-im-internet.de/hebg_2020/BJNR175910019.html], [https://www.gesetze-im-internet.de/hebstprv/BJNR003900020.html]. This interprofessional focus needs the development of courses on complex care situations in which midwifery students are taught and supervised in alongside students from other degree programmes (such as medicine, nursing) [16]. However, the implementation of these requirements is a major challenge especially for universities, both in terms of planning and capacity. Unfortunately, joint courses often fail due to the availability of room sizes or the differing organisation of semester schedules. 

As future healthcare experts for women of childbearing age, student midwives must also be educated in women’s health and related research, including the ability to identify and address health inequalities and their impact on vulnerable clients. In doing so, students benefit from a strong grounding in sociology and gender studies.

### 3.2. Practice

In comparison with vocational training, clinical placements in academic training were reduced from 3,000 hours to 2,200 hours. However, the proportion of academic clinical instruction by a mentor midwife was increased to 25%. Nevertheless, in some federal states, a reduced volume of no less than 15% clinical instruction is possible until 2030. Clinical placements cover the following areas: maternity and antenatal ward, labour ward, neonatal unit and gynaecological department. In addition, the attendance of clinical placements must be documented across semesters and practical experience is linked to theory in appropriate modules respectively (Appendix 3 of the Midwifery Act). 

The organisation of clinical placements for midwifery students is challenging due to a lack of cooperating practice partner hospitals. Furthermore, clinical placements must take place with academically trained mentors to achieve skills at the level of DQR 6. However, the requirements in practice vary considerably [14]. Universities of applied sciences are particularly well equipped to compensate for different requirements in clinical practice, as they have many years of experience in the implementation of dual study programmes with diverse clinical partners [5]. Differences in the quality of clinical advice/guidance between the different collaborating hospital partners are to be expected. The different levels of care at hospitals also lead to differences in clinical guidance. For instance, students placed in level 1 hospitals with perinatal centres will gain a deeper insight into the care of pregnant women with risk factors and pathologically complex pregnancy and childbirth. Whereas placements in level 3 hospitals allow student midwives to be more independent in the care of physiological processes. However, it would be beneficial for students to become familiar with maternity units at different levels of care as part of their training. This pathway would enable future midwives to become proficient in both independent midwifery care for physiological processes and interdisciplinary maternity care for complex care. Expertise in the care of both physiological and pathological cases is explicitly required by law [https://www.gesetze-im-internet.de/hebg_2020/BJNR175910019.html], [https://www.gesetze-im-internet.de/hebstprv/BJNR003900020.html].

A current challenge is the shortage of trained mentor midwives needed to meet the statutory requirement for 25% of individual clinical instruction. Another challenge in many regions is the lack of cooperating hospitals as practice partners. Consequently, the number of clinical placements is not always in agreement with the number of students. However, securing the next generation of midwives can only be achieved if the qualification of midwives in the federal states is seen as a necessary common task of universities, hospitals and practice partners in community settings.

### 3.3. Theory-practice transfer

A central aspect of dual degree programmes is the theory-practice transfer, where theoretical components support practice and vice versa. This is achieved through by linking theoretical and clinical components. It is recommended to implement theory-practice transfer measures from the first semester onwards, where possible, and to integrate them into a longitudinal curriculum with graded skills acquisition in all semesters up to the state exam. The range of potential learning tools to facilitate theory-practice transfer is extensive and should be used in a variety of ways, depending on the availability of staff resources and the specific thematic focus of each semester. The development of these learning tools is the responsibility of academic staff in curricular teaching, as well as of clinical instructors in the field. In this context, it is important to emphasise the significance of skills training and simulation. These methods can facilitate the consolidation of both basic and complex skills in a simulated environment. Acquiring expertise in skills training and simulation is particularly relevant for students, as clinical state exam in the competence area of childbirth is obligatory to be a simulated scenario [17]. Regular practice assessments in a simulated environment, such as “Objective Structured Clinical Examination” (OSCE), can also be part of the theory-practice transfer to monitor the progress of skills acquisition at the interface between theory and practice in each semester [18]. Furthermore, clinical instructions by link lectures is required by law [https://www.gesetze-im-internet.de/hebg_2020/BJNR175910019.html], [https://www.gesetze-im-internet.de/hebstprv/BJNR003900020.html]. The methods used for theory-practice transfer are planned and implemented by the university's teaching staff at the clinical learning sites. As there are no specifications regarding scope and content of teaching and learning methods neither in the Midwifery Act and nor by the government authorities of the federal states, methods and educational tools can be individually designed by each university. These methods and tools could include supervised clinical visits, development of care plans, case studies and both reflective writing and discussions. Finally, the regular integration of academic staff and other practitioners (midwives, nurses and doctors) in clinical instruction within the degree programme is essential. A functional collaboration can be achieved through regular meetings with cooperating hospitals, university-initiated skills and simulation training, and clinical visits by the university’s link lecturers. Prior to providing guidance to students in a clinical setting, it is essential that clinical mentors are familiar with the theory content and are also involved in skills training and simulations, as well as in the design and implementation of practical assessments.

## 4. Tests

The module-based curriculum developed by the faculties of midwifery science serves as the foundation for all theoretical and practical skills and simulation courses as well as in the clinical element of the programme. As mandated by §§19-22 HebG, hospital partners work in tandem with faculties to adjust the students’ clinical placement experiences to the theoretical curriculum. Each module is completed with a statutory module assessment. The format of this assessment, the number of subtests including their duration are part of the university’s specific curriculum and are specified in the Study and Exam Regulations. They are also listed in the respective module handbooks. Due to the regulatory authority of the federal states in Germany and the 'freedom of teaching and research'-rule at German institutions of higher education, there is a certain degree of heterogeneity with regard to teaching and assessment formats and the scope of exams. This however makes it difficult to compare bachelor degrees of midwifery science across institutes (see attachment 1 , table S1). 

As per §21 of the HebStPrV [https://www.gesetze-im-internet.de/hebstprv/BJNR003900020.html], the written exam should emphasize specific competencies outlined in attachment 1 , table S2. However, this narrow focus on individual competencies contradicts the purpose of both the bachelor's and state qualifications, which aim to demonstrate an overall professional competence as a midwife. It also contrasts with the intended direction of the professional exams, which seek to establish a broader, science-based competency profile for a Bachelor of Science in Midwifery.

In the simulated part of the state exam, students are required to demonstrate all midwifery competencies in relation to antenatal, intrapartum and postpartum care, and breastfeeding support, as outlined in Appendix 1 of the HebStPrV. This simulated exam is the only opportunity for a comprehensive assessment of the competence profile of a Bachelor of Science. The simulation exam is compulsory, especially for the competence are “intrapartum care”. It replaces the former practical exam, which was carried out during real and therefore unpredictable labour. This change was inevitable for a number of reasons, most notably ethical concerns for the birthing woman and the difficulty of making examination situations comparable [19]. In addition, the revised assessment format now considers community-based midwifery, a previously neglected aspect, as a potential setting for the practical state exam, a shift from the previous exam guideline.

The practical part of the state exam is particularly challenging for universities [17]. The need to establish a well-equipped skills/simulation laboratory with modern technical and digital equipment is central to these challenges. However, the substantial financial investment required for this often exceeds university budgets and typically relies on initial funding from federal states. Moreover, ongoing financial support is essential for supplies, administration, IT expertise, and training and deployment of acting clients in simulation. Additionally, the teaching team needs to be trained in continuous didactic planning and professional delivery of skills lab training throughout the study programme. This, in turn, involves additional costs for the faculties or departments in which midwifery programmes are located. It is therefore crucial that institutions offering bachelor’s degrees in midwifery science, which are responsible for producing experts in maternal, child, and family healthcare, receive adequate funding to support these new structures.

The pedagogical and didactic design of midwifery studies is influenced by the requirements of professional law with regard to the state examination of midwives. Therefore, professional skills training must be integrated throughout the curriculum to enable students to practise in the examination format of simulation and skills orientation. Didactic decisions need to be made about learning situations and the use of models with varying degrees of digitalisation and fidelity, depending on the competency objectives and the complexity of the subject matter. Furthermore, it is recommended that all assessments for the State Exam for Midwives be based on a comprehensive competency profile that goes beyond the competency requirements of the HebStPrV [https://www.gesetze-im-internet.de/hebstprv/BJNR003900020.html]. This is the only way to ensure that the full competence of a qualified midwife at B.Sc. level is tested in the professional exams, particularly the ability to act in a scientifically sound manner (competence II) and to promote the autonomy (independence and self-determination) of women (competence III, see attachment 1 , table S2).

According to § 4 HebG [https://www.gesetze-im-internet.de/hebg_2020/BJNR175910019.html] and HebStPrV [https://www.gesetze-im-internet.de/hebstprv/BJNR003900020.html], the focus must extend beyond midwifery care/midwifery activities to interdisciplinary maternity care provided by doctors and other allied/associated healthcare professions (for example nurses). However, the implementation of interdisciplinary teaching/learning settings is hampered by the different learning content, competence objectives and assessment procedures of the respective professional groups or study programmes. Given the limited staffing resources available, the didactic networking of different study programmes represents an additional challenge [20]. It is apparent that the success of this endeavour depends on universities having sufficient human and financial resources.

## 5. Conclusion

The implementation of an academic training for the midwifery profession in Germany offers substantial potential for the development of the discipline and the optimisation of maternity care for women, their children and families. However, it also represents a significant challenge for midwifery science programmes. Given the structural and professional heterogeneity observed in the different study locations, it is essential that all academic institutes involved in midwifery programmes network to promote the long-term development of the discipline of midwifery. 

The Midwifery Science Committee (AHW) of the DACH Association for Medical Education (GMA), established in June 2022, facilitates networking between locations and the exchange of experiences on teaching, learning and assessment formats. Furthermore, it develops standardised competence frameworks and a core curriculum, as well as quality criteria and standards for midwifery science programmes. The professional development of both midwifery and midwives can only be successful if all stakeholders work together and pool their expertise.

## Notes

### Authors’ ORCIDs

- Claudia F. Plappert:[0000-0003-1896-3959]
- Nicola H. Bauer: [0000-0002-7351-0832]
- Kirsten Dietze-Schwonberg: [0000-0001-7649-4754]
- Melita Grieshop: [0009-0001-6243-2306]
- Annette Kluge-Bischoff: [0000-0002-3317-3501]
- Birgit-Christiane Zyriax: [0000-0002-5377-5956]
- Sabine Striebich: [0000-0002-0626-3552]

### Adopted

The position paper was adopted by the GMA executive board at May 7 2024.

## Acknowledgements

The authors of the position paper would like to thank the following experts for their advice during the completion of the manuscript:

- Prof. Anne Wiedermann, Chair of Midwifery Science, Faculty of Interdisciplinary Studies at Landshut University of Applied Sciences
- Prof. Dr. Susanne Grylka, Head of Research Institute for Midwifery Science and Reproductive Health at the Zurich University of Applied Sciences ZHAW
- Dr. Astrid Krahl, Head of MSc Midwifery at the Institute of Midwifery, Department of Health, ZHAW Zurich University of Applied Sciences

The authors would like to thank Janice Hill (University of Tübingen) and Emine Babac (Martin Luther University Halle-Wittenberg) for translating the manuscript.

## Competing interests

The authors declare that they have no competing interests. 

## Supplementary Material

Supplementary tables
