# Supplementary material for: Academic education of midwives in Germany (part 1): Requirements for bachelor of science programmes in midwifery education. Position paper of the Midwifery Science Committee (AHW) in the DACH Association for Medical Education (GMA)
Source: GMS J Med Educ. 2024 Jun 17;41(3):Doc33. doi: 10.3205/zma001688 (PMC11310784; doi:10.3205/zma001688)
Supplement: Supplementary tables [file JME-41-33-s-001.pdf]

Anhang 1: Ergänzende Tabellen

Tabelle S1: *Kompetenzen von B.SC. Hebammen gemäß HebStPrV 2020 [https://www.gesetze-im-internet.de/hebstprv/BJNR003900020.html] Anlage 1*

I.	Selbstständige und evidenzbasierte Förderung und Leitung physiologischer Prozesse während Schwangerschaft, Geburt, Wochenbett und Stillzeit. Erkennen von Risiken und Regelwidrigkeiten bei der Frau und dem Kind sowie Gewährleistung einer kontinuierlichen Hebammenversorgung unter Hinzuziehung der erforderlichen ärztlichen Fachexpertise.
II.	Wissenschaftsbasierte Planung, Organisation, Durchführung, Steuerung und Evaluation auch von hochkomplexen Betreuungsprozessen unter Berücksichtigung von Wirtschaftlichkeit, Effektivität, Qualität, Gesundheitsförderung und Prävention während Schwangerschaft, Geburt, Wochenbett und Stillzeit
III.	Förderung der Selbstständigkeit der Frauen und Wahrung ihres Rechts auf Selbstbestimmung während Schwangerschaft, Geburt, Wochenbett und Stillzeit unter Einbezug ihrer Lebenssituation, ihrer biographischen Erfahrungen sowie von Diversitätsaspekten unter Beachtung der rechtlichen Handlungspflichten
IV.	Personen- und situationsorientierte Kommunikation während des Betreuungsprozesses
V.	Verantwortliche Gestaltung des intra- und interprofessionellen Handelns in unterschiedlichen systemischen Kontexten, Weiterentwicklung der hebammenspezifischen Versorgung von Frauen und ihren Familien sowie Mitwirkung an der Entwicklung von Qualitäts- und Risikomanagementkonzepten, Leitlinien und Expertenstandards
VI.	Reflexion und Begründung des eigenen Handelns unter Berücksichtigung der rechtlichen, ökonomischen und gesellschaftlichen Rahmenbedingungen und berufsethischen Werthaltungen und Einstellungen sowie Beteiligung an der Berufsentwicklung

Anhang 1 zu Plappert CF, Bauer NH, Dietze-Schwonberg K, Grieshopf M, Kluge-Bischoff A, Zyriax BC, Striebich S. *Academic education of midwives in Germany (part 1): Requirements for bachelor of science programmes in midwifery education.* GMS J Med Educ. 2024;41(3):Doc33. DOI: 10.3205/zma001688

Tabelle S2: *Prüfungsformate im Hebammenstudium*

Prüfungsform	Prüfungs-format	Beschreibung
schriftliche Prüfung	Klausur	Multiple-Choice-Prüfung (Antwort-Wahl-Verfahren)<br>Multiple-Choice-(MC)-Prüfungen werden auch als Prüfungen nach dem Antwort-Wahl-Verfahren bezeichnet. Zu jeder Frage soll aus einer Auswahl an vorgegebenen Antworten die richtige ausgesucht werden. Die Erstellung von geeigneten Fragen braucht Zeit und Erfahrung. Die Korrektur hingegen ist schnell möglich [29].<br>Gemeinschaftlich gestellte Klausur<br>Die gemeinschaftlich gestellte Klausur ist ein schriftliches Prüfungsformat, bei dem die Studierenden in einer vorgegebenen Zeit Aufgaben bearbeiten. Die Aufgaben sind verschiedenen Teilbereichen zugeordnet und werden von den jeweils verantwortlichen Lehrenden gestellt [30].<br>Fallbasierte Klausur<br>Kognitive Lehr-/Lernziele, die auf höheren taxonomischen Stufen angesiedelt sind, benötigen Prüfungsformate, die über das primäre Reproduzieren und Anwenden von Faktenwissen hinausgehen. Fallbasierte Klausuren ermöglichen in diesem Kontext, das Erreichen höherwertiger Lernergebnisse zu überprüfen [31].
	E-Klausur	Eine E-Klausur ist das computergestützte Pedant zur papierbasierten Klausur und ermöglicht die elektronische Durchführung und auch teilweise Auswertung. Dieses Klausurformat kann in vielen Bereichen kompetenzorientierter prüfen als andere schriftliche und mündliche Prüfungen [32].
	Hausaufgabe/ Seminararbeit	Die Hausarbeit hat zum Ziel, dass Studierende eine fachliche Fragestellung schriftlich bearbeiten und beantworten. Dies hat auf Grundlage des fachlichen Wissens und einer Reihe formaler und sprachlicher Vorgaben zu geschehen. Sie verlangt viel Eigenständigkeit auf mehreren Ebenen [33].
	Reflexions-bericht	Reflexionsberichte entstehen im Nachgang zu von den Studierenden selbst erlebten berufspraktischen Handlungssituationen. Reflexionsberichte werden, nachdem die Studierenden eine berufspraktische Handlung selbst durchgeführt haben, erstellt und enthalten eine kompakte Beschreibung der Situation sowie eine anschließende Reflexion der gemachten Erfahrungen, erkannten Lernherausforderungen und Entwicklungsfelder. Sie stellen die konstruktive Verbindung zwischen Lernzielen, studentischer Aktivität und Prüfung dar [34].
Portfolio-Prüfung	Portfolio	Die Portfolio-Prüfung ist ein personalisiertes Prüfungsformat. Hierbei wird die Reflexionsfähigkeit von Studierenden bezogen auf die eigene Entwicklung im Hinblick auf einen konkreten Reflexionsgegenstand in einem festgelegten und im Portfolio dokumentierten Zeitraum geprüft. Im sich anschließenden Prüfungsgespräch wird die erreichte Reflexionstiefe bewertet [35].
	E-Portfolio	Eine E-Portfolio-Prüfung ist eine formativ-summative Prüfung. Sie besteht aus einer Reihe von kumulativen Vorleistungen (Artefakten) und einer abschließenden mündlichen oder (seltener) schriftlichen E-Portfolio-Prüfung, in der auf ausgewählte Artefakte Bezug genommen wird [36].

Anhang 1 zu Plappert CF, Bauer NH, Dietze-Schwonberg K, Grieshopf M, Kluge-Bischoff A, Zyriax BC, Striebich S. *Academic education of midwives in Germany (part 1): Requirements for bachelor of science programmes in midwifery education.* GMS J Med Educ. 2024;41(3):Doc33. DOI: 10.3205/zma001688

mündliche Prüfung	Mündliche Einzelprüfung	In der mündliche Prüfung, auch als Prüfungsgespräch bezeichnet, erfolgt eine Befragung zu den Lernzielen und -inhalten des Studiums, eines Moduls oder einer einzelnen Lehrveranstaltung [37].
praktische Prüfung	Objektive strukturierte praktische/ klinische Prüfung	Eine objektive strukturierte praktische/klinische Prüfung (engl. Objective Structured Practical/Clinical Examination [OSPE/OSCE]) dient der Kontrolle klinisch-praktischer Fertigkeiten in medizinischen Fächern. Mittels klinisch-praktischer Prüfungsstationen können Kompetenzen wie klinische Handlungsentscheidungen, kommunikative Fertigkeiten bis hin zu ethischen Haltungen abgebildet und geprüft werden [38].
	Mini-Clinical Evaluation Exercise	Unter einer „Mini-Clinical Evaluation Exercise“ versteht man eine arbeitsplatzbasierte Bewertung, bei der die oder der Studierende während eines 10- bis 20minütigen Patientenkontakts beim Durchführen alltäglicher Maßnahmen vom Prüfer oder der Prüferin direkt beobachtet wird (z.B. bei Anamnesen oder körperlichen Untersuchungen). Der Fokus liegt auf der Kommunikation oder der Untersuchung. Es folgt ggf. eine Fallzusammenfassung, eine Selbsteinschätzung und im Anschluss ein strukturiertes schriftliches oder mündliches Feedback (5 bis 10 Minuten) durch den Prüfer oder die Prüferin. Fremd- und Selbsteinschätzung können ggf. verglichen werden, Stärken und Schwächen werden besprochen und weitere Lernziele werden gemeinsam abgeleitet [39].
	Direct Observation of Procedural Skills	Die “Direct Observation of Procedural Skills“ ist eine arbeitsplatzbasierte Bewertung, bei der die oder der Studierende bei der Durchführung häufig vorkommender klinisch-praktischer Maßnahmen unterschiedlicher Komplexität von einem Prüfer oder einer Prüferin direkt beobachtet wird. Der Fokus liegt auf den „manuell technischen Fertigkeiten“. Zudem kann die Interaktion mit Patient*innen oder Kolleg*innen beobachtet und professionelles Verhalten mitbewertet werden [39].
